# Decreased SUV39H1 at the promoter region leads to increased CREMα and accelerates autoimmune response in CD4^+^ T cells from patients with systemic lupus erythematosus

**DOI:** 10.1186/s13148-022-01411-7

**Published:** 2022-12-20

**Authors:** Shuangyan Luo, Huilin Zhang, Yuming Xie, Junke Huang, Danhong Luo, Qing Zhang

**Affiliations:** 1 Hunan Key Laboratory of Medical Epigenomics, Department of Dermatology, The Second Xiangya Hospital, Central South University, #139 Renmin Middle Rd, Changsha, 410011 Hunan People’s Republic of China; 2 Clinical Nursing Teaching and Research Section, The Second Xiangya Hospital, Central South University, #139 Renmin Middle Rd, Changsha, 410011 Hunan People’s Republic of China; 3 Department of Dermatology, The Fifth People’s Hospital of Hainan Province, #49 Longkun South Rd, Haikou, 570206 Hainan People’s Republic of China

**Keywords:** Systemic lupus erythematosus, CREMα, SUV39H1, H3K9me3, Set1, H3K4me3, DNA methylation, DNMT3a

## Abstract

**Background:**

Overproduction of cAMP-responsive element modulator α (CREMα) in total T cells from patients with systemic lupus erythematosus (SLE) can inhibit IL-2 and increase IL-17A. These ultimately promote progression of SLE. This study aims to investigate the expression of CREMα in SLE CD4^+^ T cells and find out the mechanisms for the regulation of CREMα in SLE CD4^+^ T cells.

**Results:**

CREMα mRNA was overexpressed in CD4^+^ T cells from SLE patients. The levels of histone H3 lysine 9 trimethylation (H3K9me3) and suppressor of variation 3–9 homolog 1 (SUV39H1) at the CREMα promoter of SLE CD4^+^ T cells were markedly decreased. Down-regulating SUV39H1 in normal CD4^+^ T cells elevated the levels of CREMα, IL-17A, and histone H3 lysine 4 trimethylation (H3K4me3) in the CREMα promoter region, and lowered IL-2, H3K9me3, DNA methylation, and DNA methyltransferase 3a (DNMT3a) enrichments within the CREMα promoter, while no sharp change in SET domain containing 1 (Set1) at the CREMα promoter. Up-regulating SUV39H1 in SLE CD4^+^ T cells had the opposite effects. The DNA methylation and DNMT3a levels were obviously reduced, and H3K4me3 enrichment was greatly increased at the CREMα promoter of CD4^+^ T cells from SLE patients. The Set1 binding in the CREMα promoter region upgraded significantly, and knocking down Set1 in SLE CD4^+^ T cells alleviated the H3K4me3 enrichment within this region, suppressed CREMα and IL-17A productions, and promoted the levels of IL-2, CREMα promoter DNA methylation, and DNMT3a. But there were no obviously alterations in H3K9me3 and SUV39H1 amounts in the region after transfection.

**Conclusions:**

Decreased SUV39H1 in the CREMα promoter region of CD4^+^ T cells from SLE patients contributes to under-expression of H3K9me3 at this region. In the meantime, the Set1 binding at the CREMα promoter of SLE CD4^+^ T cells is up-regulated. As a result, DNMT3a and DNA methylation levels alleviate, and H3K4me3 binding increases. All these lead to overproduction of CREMα. Thus, the secretion of IL-2 down-regulates and the concentration of IL-17A up-regulates, ultimately promoting SLE.

## Background

Systemic lupus erythematosus (SLE) is a chronic autoimmune disease involving multiple organs and systems, seriously affecting patients’ health and live [1]. The basic pathogenesis of SLE is over-activated CD4^+^ T cells stimulate B cells, leading to over-secretion of autoantibodies. The progression of SLE involves many immune-related genes, in which cAMP-responsive element modulator α (CREMα) plays an important role. Studies find that the CREMα level of total T cells in patients with SLE significantly elevates, and the activity of its promoter region positively correlates with the SLE disease activity index (SLEDAI) [2, 3]. Increased CREMα inhibits the transcription of TCR/CD3 *ζ* chain, thus hindering its termination of T cells response [4, 5]. It also inhibits antigen-presenting cell molecule CD86, transcription factor c-fos, and Notch signaling pathway molecule Notch-1 to promote the onset of SLE [6–8]. Importantly, overexpression of CREMα leads to deficiency in IL-2 [3, 6, 9, 10] and augment of IL-17A [2, 7–9, 11] at the same time, resulting in a variety of inflammatory reactions, and ultimately the development and progression of SLE. However, the quantity and regulations of CREMα in SLE CD4^+^ T cells remain unclear. Given the important role of CREMα in SLE, the investigation on the expression and regulatory mechanisms of CREMα in CD4^+^ T cells of SLE can further clarify the pathogenesis and provide new effective targets for treating SLE.

Recently, the role of epigenetics in the progression of SLE has received great attention. Epigenetic mechanisms mainly involve DNA methylation, histone modification, chromatin remodeling, and regulation of noncoding RNA [12–14]. Studies have shown that H3 lysine 9 trimethylation (H3K9me3) [15–18] and DNA methylation [19–22] lead to transcriptional inhibition, while H3 lysine 4 trimethylation (H3K4me3) is related to transcriptional activation [23, 24]. Of these, H3K9me3, as one of the most common histone modifications leading to transcriptional repression, has been a research hotspot. Studies have found that H3K9me3 combines with heterochromatin protein-1 (HP-1) to form heterochromatin, resulting in a dense chromatin structure, which prevents transcription factors from entering chromatin and playing roles, thus promoting gene silencing [20, 25–27]. Moreover, many studies have proved that H3K9me3 also recruits various DNA methyltransferases and promotes DNA methylation [19, 20, 28–30]. H3K9me3 also reduces the H3K4 methylation level by rejecting H3K4 methyltransferases or recruiting H3K4 demethylases [31–33]. It is well known that the suppressor of variation 3–9 homolog 1 (SUV39H1) and the suppressor of variation 3–9 homolog 2 (SUV39H2) are mainly histone methyltransferases (HMTs) that catalyze the trimethylation of H3K9 [21, 34–36].

In our study, we first confirmed that CREMα was overexpressed in SLE CD4^+^ T cells. Whereafter, in order to understand further the mechanism of CREMα overactivation, we started the research from an epigenetic point of view. The DNA methylation enrichment at the CREMα promoter of total T cells from SLE patients has been proved to be sharply lowered [2], and DNA methyltransferase 3a (DNMT3a) within the CREMα promoter region of SLE CD4^+^ T cells is greatly attenuated, while the H3K4me3 and SET domain containing 1 (Set1, an important H3K4 methyltransferase) bindings in this region are strikingly elevated [37]. As mentioned before, H3K9me3 can affect the levels of DNA methylation [19, 20, 28–30] and H3K4 methylation [31–33]. According to these clues, we examined the H3K9me3 binding in the CREMα promoter region, and verified it was decreased in CD4^+^ T cells of SLE relative to normal controls. Furthermore, SUV39H1 enrichment at the CREMα promoter region of SLE CD4^+^ T cells was alleviated greatly, while SUV39H2 had no profoundly change. Inhibiting the expression of SUV39H1 by SUV39H1-siRNA transfection in CD4^+^ T cells of normal controls resulted in down-regulation of IL-2, reductions of SUV39H1, H3K9me3, DNA methylation, and DNMT3a at the CREMα promoter, and over expression of CREMα and IL-17A. In this region, the H3K4me3 level elevated, while Set1 had no significant change after transfection. The opposite effects were observed when SLE CD4^+^ T cells were transfected with the SUV39H1-overexpressing plasmid. The DNA methylation and DNMT3a levels at the CREMα promoter of SLE CD4^+^ T cells were profoundly deficient, and the H3K4me3 enrichment in the region was sharply increased. We further proved that the Set1 binding was upgraded at the CREMα promoter in CD4^+^ T cells from SLE patients. Knocking down Set1 by Set1-siRNA in SLE CD4^+^ T cells contributed to up-regulation of DNA methylation and DNMT3a at the CREMα promoter, and overproduction of IL-2, while Set1 and H3K4me3 enrichments within the region were attenuated, and CREMα and IL-17A abundances were suppressed. However, there were no statistical alterations in H3K9me3 and SUV39H1 enrichments at the CREMα promoter after transfection. These results reveal the role that SUV39H1 plays in the pathogenesis of SLE for the first time ever, and are expected to provide new ideas for SLE treatment.

## Methods

### Subjects

A total of 20 patients with SLE and 20 age- and sex-matched normal controls were recruited in this study. All patients (age: 28.40 ± 5.73 years) were enrolled from the outpatient dermatology clinic and inpatient wards of the Second Xiangya Hospital, Central South University. The relevant patient profiles are listed in Table 1. All patients fulfilled the SLE classification criteria of the American College of Rheumatology (ACR) [38]. Their disease activities were evaluated using the SLEDAI [39]. Healthy controls (age: 29.55 ± 5.42 years) were enrolled from students and staff of the Central South University, and the relevant profiles are listed in Table 2. This study was approved by the Human Ethics Committee of the Second Xiangya Hospital, Central South University, and written informed consent was obtained from each participant prior to inclusion in the study.

**Table 1:** Patient profiles

Patient	Gender	Age (years)	SLEDAI	Medications
1	Female	21	5	Pred 30 mg/d
2	Female	26	8	Pred 40 mg/d, TG 30 mg/d
3	Female	25	6	Pred 20 mg/d, HCQ 0.2 g/d
4	Female	40	11	Pred 40 mg/d, CsA 150 mg/d
5	Female	34	9	Pred 40 mg/d
6	Female	27	3	None
7	Female	25	2	Pred 5 mg/d
8	Female	20	8	Pred 20 mg/d, MMF 1.5 g/d, HCQ 0.2 g/d
9	Male	25	12	None
10	Female	36	14	Pred 15 mg/d
11	Female	35	8	Pred 40 mg/d
12	Female	33	3	Pred 5 mg/d, HCQ 0.2 g/d
13	Female	25	16	Pred 50 mg/d, MMF 1.5 g/d
14	Male	24	2	Pred 30 mg/d
15	Female	23	12	Pred 35 mg/d, TG 30 mg/d
16	Female	36	16	Pred 40 mg/d, TG 30 mg/d, HCQ 0.2 g/d
17	Female	32	6	None
18	Female	27	4	None
19	Female	23	15	Pred 50 mg/d, CsA 150 mg/d
20	Female	31	7	Pred 30 mg/d, HCQ 0.2 g/d

*SLEDAI*, systemic lupus erythematosus disease activity index; *Pred*, prednisone; *TG*, tripterygium glycoside; *HCQ*, hydroxychloroquine; *CsA*, cyclosporin A; and MMF: mycophenolate mofetil

**Table 2:** Normal control profiles

Normal control	Gender	Age(years)
1	Female	26
2	Female	23
3	Female	25
4	Female	28
5	Female	27
6	Male	34
7	Female	37
8	Female	36
9	Female	27
10	Female	23
11	Female	25
12	Female	24
13	Female	26
14	Female	41
15	Female	36
16	Male	27
17	Female	28
18	Female	35
19	Female	35
20	Female	28

### Cell preparation

Venous peripheral blood was withdrawn from each healthy control and patient and preserved in heparin. Peripheral blood mononuclear cells were then separated by density gradient centrifugation (GE Healthcare). CD4^+^ T cells were isolated by positive selection using human CD4 beads, following the manufacturer’s protocol (Miltenyi). The purity of the CD4^+^ T cells was evaluated by flow cytometry and was generally higher than 95%.

### RNA isolation, cDNA synthesis, and quantitative PCR (qPCR)

Total RNA from CD4^+^ T cells was extracted using TRIzol reagent (Thermo Fisher Scientific), and complementary DNA (cDNA) was synthesized from 1 μg of total RNA using a miScript II Reverse Transcription Kit (Qiagen), following the manufacturer’s protocols. The reaction mixture of qPCR contained 10 μL SYBR Premix Ex Taq II (TaKaRa), 2 μL cDNA, 10 μM sense primer, and 10 μM antisense primer to a final volume of 20 μL. The amounts of mRNA were normalized to β-actin. The primers used in the study were as follows: for CREMα, 5′-GAAACAGTTGAATCCCAGCATGATGGAAGT-3′ (forward) and 5′- TGCCCCGTGCTAGTCTGATATATG-3′ (reverse); for β-actin, 5′-CGCGAGAAGATGACCCAGAT-3′ (forward) and 5′-GCACTGTGTTGGCGTACAGG-3′ (reverse). All results were measured thrice.

### Chromatin immunoprecipitation (ChIP) assay and qPCR

The ChIP assay for histone methylation was performed according to the protocol provided in the ChIP kit (Millipore) as described previously, and input DNA (total chromatin) and normal rabbit IgG were used as endogenous control and negative control, respectively [40]. The anti-H3K9me3 antibody and anti-H3K4me3 antibody were provided by Millipore, and the anti-SUV39H1 antibody, anti-SUV39H2 antibody, anti-Set1 antibody, and anti-DNMT3a antibody were purchased from Abcam. QPCR was performed with an ABI Prism 7500 instrument (Thermo Fisher Scientific), and the level of deposited DNA was calculated using the standard curve method. The primers of CREMα promoter were as follows: 5′-TGGGGAGATAGAGGTTGCAG-3′ (forward) and 5′-CGCCAGAAATCCAATGACTT-3′ (reverse). All reactions were run three times.

### Transfection

Control-siRNA, SUV39H1-siRNA, Set1-siRNA, pcDEF3 blank plasmid, and pcDEF3-SUV39H1-expressing plasmid were all designed and synthesized at Guangzhou RiboBio in China. CD4^+^ T cells were transfected using a nucleofector and a Human T Cell Nucleofector kit (Amaxa), following the protocol provided by the manufacturer. In brief, CD4^+^ T cells were enriched, resuspended in 100 µL Human T Cell Nucleofector solution, and mixed with siRNA or plasmid. The mixture was subsequently electrotransfected using the program V-024 in the nucleofector. The transfected T cells were then cultured in human T cell culture medium at 37 °C with 5% CO_2_. 24 h after transfection, the cells were stimulated with 5.0 μg/mL anti-CD3 and 5 μg/mL anti-CD28 antibodies for 48 h. Whereafter, the cells and supernatants were harvested for further analysis.

### Western blot analysis

The CD4^+^ T cells were lysed in whole-cell lysis buffer containing proteinase inhibitor (Thermo Fisher Scientific). Subsequently, the lysates were centrifuged, and the supernatants were enriched. The protein concentrations were measured by the Bradford Protein Assay (HyClone-Pierce). After denaturation, the proteins were separated by sodium dodecyl sulfate–polyacrylamide gel electrophoresis with 8% polyacrylamide gels and then transferred onto PVDF membranes (Millipore). The membranes were blocked with 5% nonfat milk in Tris-buffered saline–Tween buffer and immunoblotted with anti-SUV39H1 antibody (1:1000, Abcam), anti-CREMα antibody (1:500, Abcam), anti-Set1 antibody (1:500, Abcam), or anti-β-actin antibody (1:1000, Santa Cruz). The blots were exposed to X-ray films, and band intensities were assessed by Quantity One software (Bio-Rad). All experiments were performed in triplicate.

### Enzyme-linked immunosorbent assays (ELISA)

IL-2 and IL-17A concentrations in the supernatants of transfected CD4^+^ T cells were examined using the human IL-2 and IL-17A ELISA kits (Yuanxiang), respectively, according to the manufacturer’s instructions. The optical density values were read at 450 nm for the productions of both IL-2 and IL-17A using an ELx800 Absorbance Microplate Reader (Bio-Tek). Three replicate wells were used for each sample, and all experiments were performed thrice.

### Methylated DNA immunoprecipitation (MeDIP) assay and qPCR

The MeDIP assay was carried out following the manufacturer’s protocol (Abcam). Briefly, CD4^+^ T cells were lysed with lysis buffer, and then, DNA was sheared to fragments of 200–1000 bp using sonication. After centrifugation, the supernatants were harvested, and the sheared genomic DNA was incubated with the antibody for 5-methylcytosine or normal mouse IgG, which was used as the negative control. Subsequently, methylated DNA was released from precipitated complexes. The DNA was measured by qPCR analysis after purification, and the level was normalized to input DNA. All experiments were performed three times.

### Statistical analysis

All statistical analyses were performed using SPSS 25.0 software (SPSS Inc.). Data were expressed as mean ± standard deviation. The difference between SLE patients and normal controls were compared using the Wilcoxon rank-sum test, and the results of different transfections were compared by the paired-samples *t* test. The strength of correlations was analyzed by Pearson’s correlation coefficient. A *P* value less than 0.05 indicated a statistically significant difference.

## Results

### Up-regulated CREMα in SLE CD4^+^ T cells, and decreased H3K9me3 enrichment in the CREMα promoter region of SLE CD4^+^ T cells

**Fig. 1:**
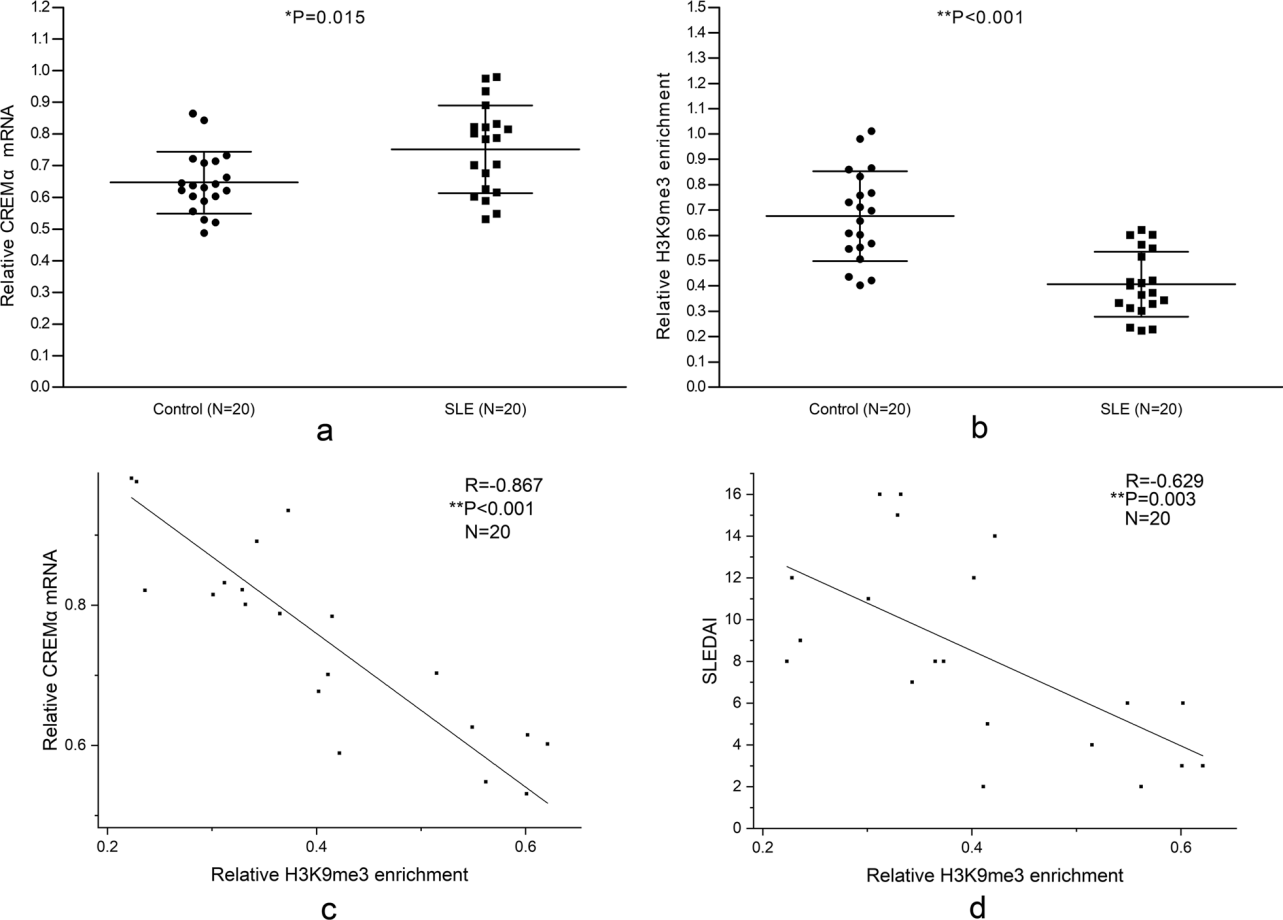
CREMα expression and H3K9me3 enrichment at the CREMα promoter in CD4^+^ T cells. **a** Relative CREMα mRNA level in normal and SLE CD4^+^ T cells was evaluated by qPCR and normalized to β-actin. **b** Relative H3K9me3 enrichment at the CREMα promoter in normal and SLE CD4^+^ T cells was measured by ChIP combined with qPCR. Input DNA was used as endogenous control, and IgG was used as negative control. **c** The correlation between H3K9me3 enrichment at the CREMα promoter and CREMα mRNA level in SLE CD4^+^ T cells. **d** The correlation between H3K9me3 enrichment at the CREMα promoter in SLE CD4^+^ T cells and SLEDAI. All reactions were run three times

The CREMα mRNA expression in CD4^+^ T cells from 20 normal controls and 20 SLE patients was compared with qPCR. The result showed that CREMα mRNA was significantly increased in SLE CD4^+^ T cells (Fig. 1a). The H3K9me3 enrichment in the CREMα promoter region of CD4^+^ T cells was measured by ChIP combined with qPCR, and it was found that the H3K9me3 enrichment in SLE patients was markedly lower than that in normal controls (Fig. 1b). In addition, the H3K9me3 enrichment at the CREMα promoter significantly negatively correlated with the CREMα mRNA level in SLE CD4^+^ T cells (Fig. 1c).

It has been proved that the activity of CREMα promoter in SLE T cells is positively correlated with SLEDAI [2, 3]. Therefore, we studied the correlation between H3K9me3 enrichment and SLEDAI and found that the H3K9me3 enrichment within the CREMα promoter of SLE CD4^+^ T cells was negatively correlated with SLEDAI (Fig. 1d).

### Down-regulated SUV39H1 binding in the CREMα promoter region of CD4^+^ T cells from SLE patients

**Fig. 2:**
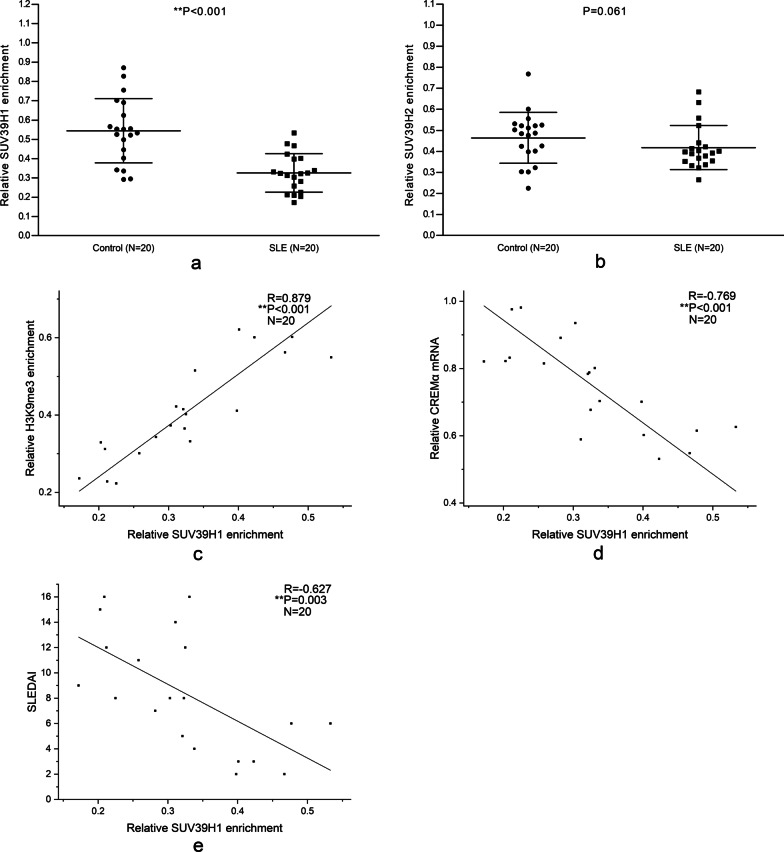
Bindings of SUV39H1 and SUV39H2 at the CREMα promoter in CD4^+^ T cells. **a**, **b** Relative SUV39H1 (**a**) and SUV39H2 (**b**) bindings at the CREMα promoter in normal and SLE CD4^+^ T cells were assessed by ChIP combined with qPCR. Input DNA was used as endogenous control, and IgG was used as negative control. **c** The correlation between SUV39H1 binding and H3K9me3 level at the CREMα promoter in SLE CD4^+^ T cells. **d** The correlation between SUV39H1 binding at the CREMα promoter and CREMα mRNA expression in SLE CD4^+^ T cells. **e** The correlation between SUV39H1 binding at the CREMα promoter in SLE CD4^+^ T cells and SLEDAI. All reactions were run three times

The levels of SUV39H1 and SUV39H2 in the CREMα promoter region of CD4^+^ T cells from the aforementioned normal controls and patients with SLE were assessed using ChIP combined with qPCR to find the reason for the decrease in the H3K9me3 enrichment at the CREMα promoter of SLE CD4^+^ T cells. The SUV39H1 level in SLE patients was intensely decreased than that in normal controls (Fig. 2a), while that of SUV39H2 had no profound difference between the two groups (Fig. 2b). Moreover, the SUV39H1 binding in the CREMα promoter region of SLE CD4^+^ T cells significantly positively correlated with the H3K9me3 enrichment (Fig. 2c) and negatively correlated with the mRNA level of CREMα (Fig. 2d) and SLEDAI (Fig. 2e).

### Inhibition of SUV39H1 expression up-regulated the levels of CREMα, IL-17A, and CREMα promoter H3K4me3, and reduced IL-2 production, CREMα promoter H3K9me3, DNA methylation, and DNMT3a amounts in normal control CD4^+^ T cells

**Fig. 3:**
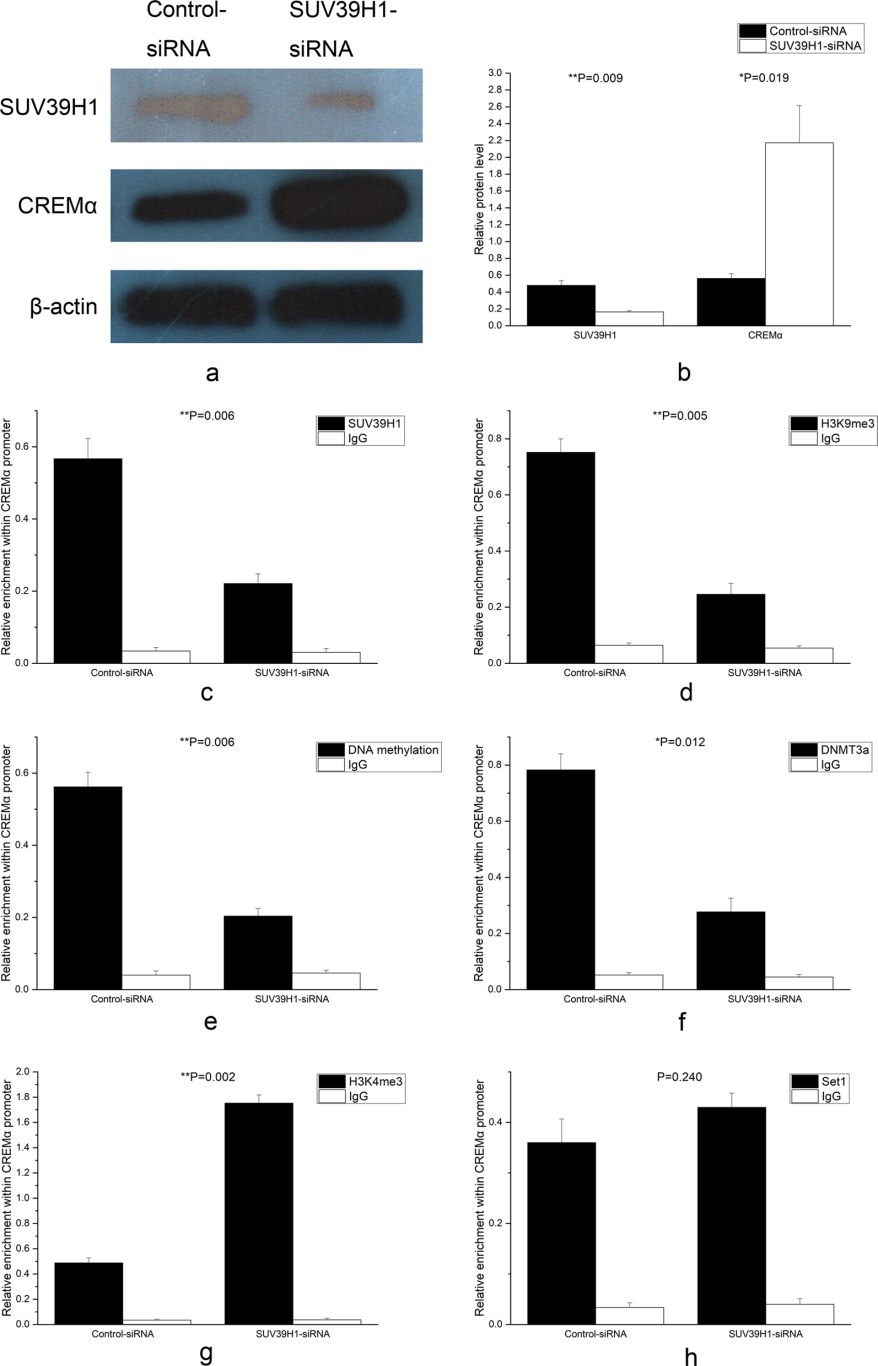
Mechanism of decreased SUV39H1 regulating CREMα expression in CD4^+^ T cells from 3 normal controls. **a**, **b** Relative SUV39H1 and CREMα protein expressions in normal CD4^+^ T cells transfected with SUV39H1-siRNA or control-siRNA were quantified by western blot analysis 72 h after transfection. β-actin was used as endogenous control. **c**, **d** Relative SUV39H1 (**c**) and H3K9me3 (**d**) enrichments at the CREMα promoter in normal CD4^+^ T cells transfected with SUV39H1-siRNA or control-siRNA were analyzed by ChIP combined with qPCR 72 h after transfection. Input DNA was used as endogenous control, and IgG was used as negative control. **e**, **f**, **g**, **h** Relative levels of DNA methylation (**e**), DNMT3a (**f**), H3K4me3 (**g**), and Set1 (**h**) at the CREMα promoter in normal CD4^+^ T cells transfected with SUV39H1-siRNA or control-siRNA were evaluated by ChIP or MeDIP combined with qPCR 72 h after transfection. Input DNA was used as endogenous control, and IgG was used as negative control. All experiments were repeated three times

CD4^+^ T cells from 3 normal controls were transfected with SUV39H1-siRNA or control-siRNA to confirm that the decrease in the SUV39H1 binding at the CREMα promoter region was the reason for the up-regulation of CREMα level in SLE CD4^+^ T cells. 72 h after transfection, the SUV39H1 protein quantity in the SUV39H1-siRNA group sharply alleviates (Fig. 3a, b), and the CREMα protein expression greatly elevated (Fig. 3a, b). Meanwhile, the levels of SUV39H1 (Fig. 3c) and H3K9me3 (Fig. 3d) in the CREMα promoter region also lowered significantly.

H3K9me3 promotes DNA methylation by recruiting DNA methyltransferases [19, 20, 28–30] and inhibits H3K4 methylation by rejecting H3K4 methyltransferases or recruiting H3K4 demethylases [31–33]. Studies confirm that the numbers of DNA methylation at the CREMα promoter of SLE total T cells and DNMT3a in the CREMα promoter region of SLE CD4^+^ T cells both down-regulate, and the quantities of H3K4me3 and Set1 within this region of SLE CD4^+^ T cells increase [2, 37]. The study explored whether these changes in SLE patients were related to alleviate H3K9me3 enrichment at the CREMα promoter. The transfected CD4^+^ T cells were examined, revealing that DNA methylation (Fig. 3e) and DNMT3a levels (Fig. 3f) at the CREMα promoter region in the SUV39H1-siRNA group greatly attenuated. The H3K4me3 enrichment in this region obviously up-regulated (Fig. 3g), but no significant difference was found in the Set1 binding (Fig. 3h).

**Fig. 4:**
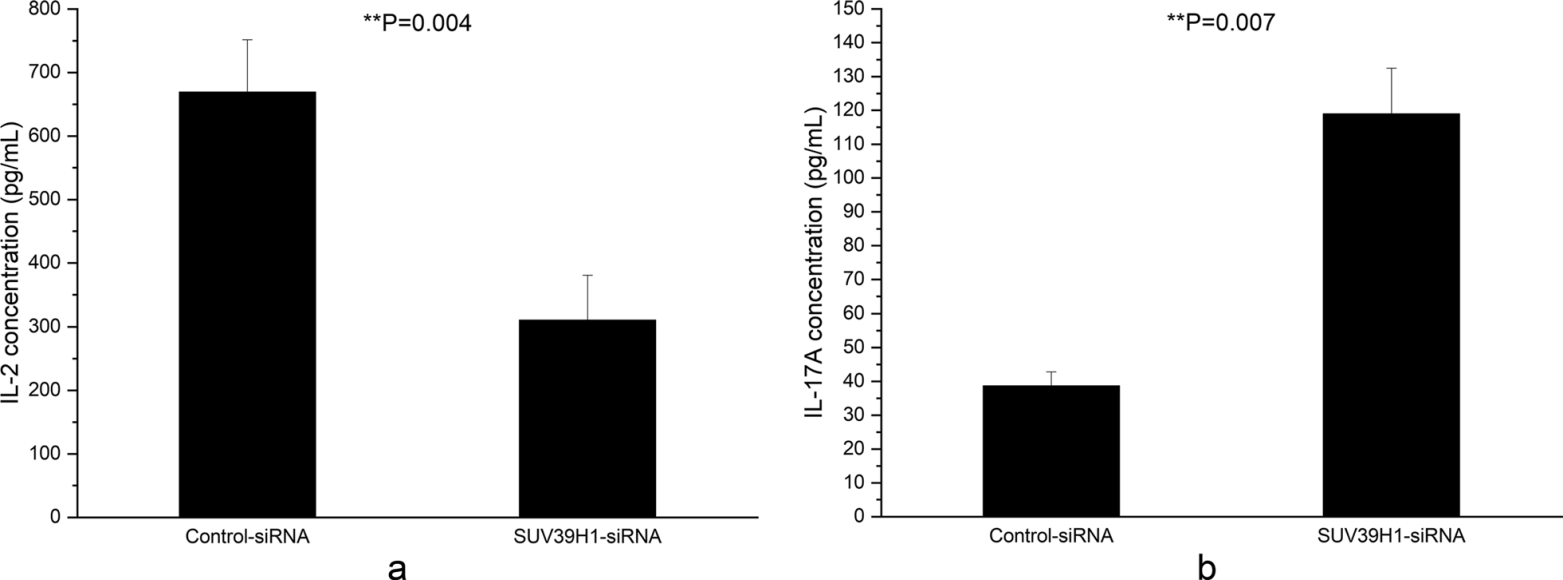
Effects of SUV39H1 down-regulation on IL-2 and IL-17A productions in CD4^+^ T cells from 3 normal controls. **a**, **b** Relative IL-2 (**a**) and IL-17A (**b**) productions in the supernatants of normal CD4^+^ T cells transfected with SUV39H1-siRNA or control-siRNA were detected by ELISA 72 h after transfection. All experiments were repeated three times

The effects of reduced SUV39H1 on IL-2 and IL-17A were also detected. After 72 h of transfection, the secretions of IL-2 and IL-17A in the supernatant were quantified using ELISA. The results showed that the IL-2 production in the supernatant of CD4^+^ T cells transfected with SUV39H1-siRNA diminished greatly (Fig. 4a), but the IL-17A over-secreted strikingly (Fig. 4b).

### Increasing the expression of SUV39H1 in SLE CD4^+^ T cells lowered the levels of CREMα, IL-17A, and CREMα promoter H3K4me3, and augmented the abundances of IL-2, H3K9me3, DNA methylation, and DNMT3a in the CREMα promoter region

**Fig. 5:**
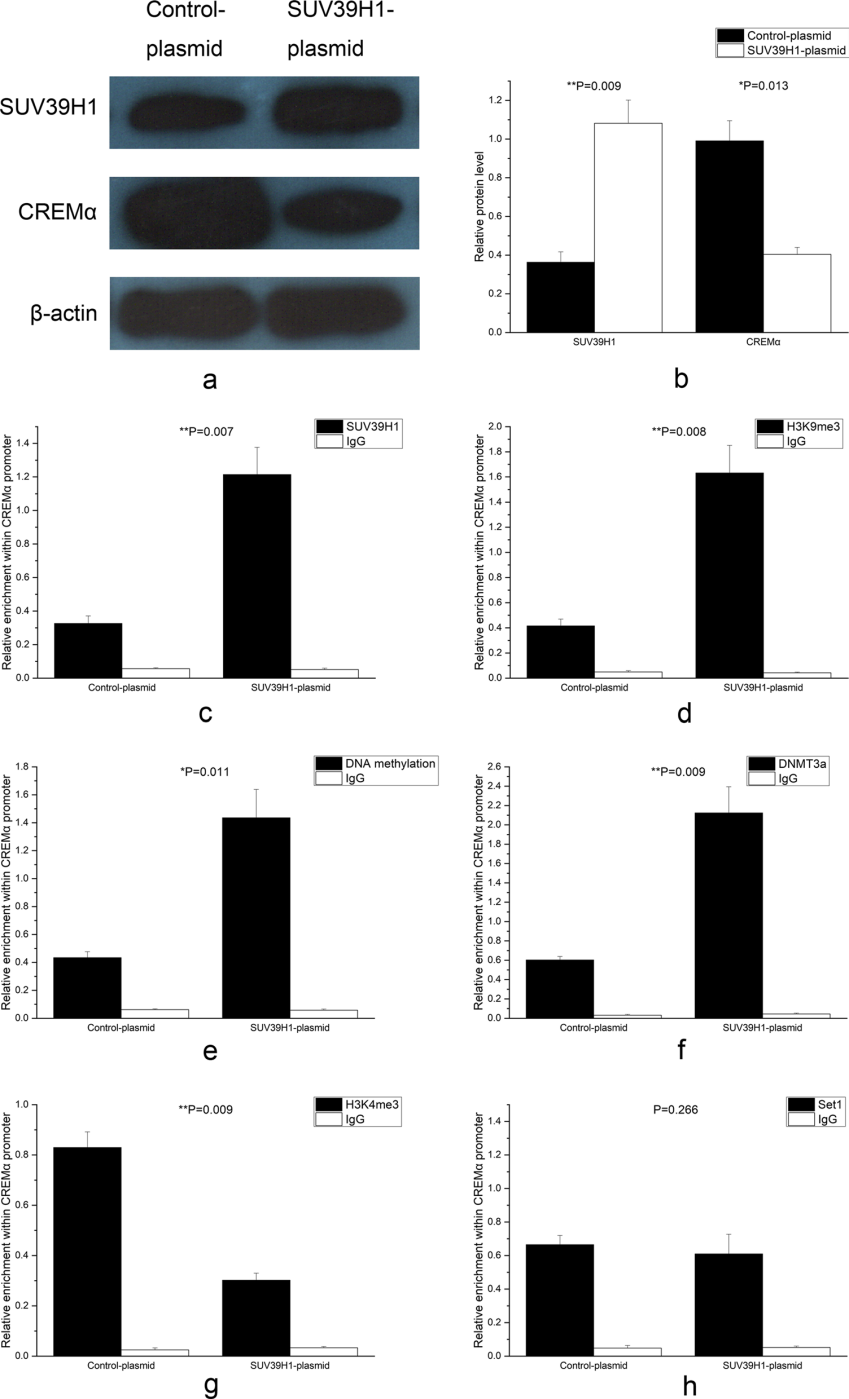
Mechanism of increased SUV39H1 regulating CREMα expression in CD4^+^ T cells from 3 SLE patients. **a**, **b** Relative SUV39H1 and CREMα protein expressions in SLE CD4^+^ T cells transfected with SUV39H1-plasmid or blank plasmid were quantified by western blot analysis 72 h after transfection. β-actin was used as endogenous control. **c**, **d** Relative SUV39H1 (**c**) and H3K9me3 (**d**) enrichments at the CREMα promoter in SLE CD4^+^ T cells transfected with SUV39H1-plasmid or blank plasmid were analyzed by ChIP combined with qPCR 72 h after transfection. Input DNA was used as endogenous control, and IgG was used as negative control. **e**, **f**, **g**, **h** Relative levels of DNA methylation (**e**), DNMT3a (**f**), H3K4me3 (**g**), and Set1 (**h**) at the CREMα promoter in SLE CD4^+^ T cells transfected with SUV39H1-plasmid or blank plasmid were evaluated by ChIP or MeDIP combined with qPCR 72 h after transfection. Input DNA was used as endogenous control, and IgG was used as negative control. All experiments were repeated three times

**Fig. 6:**
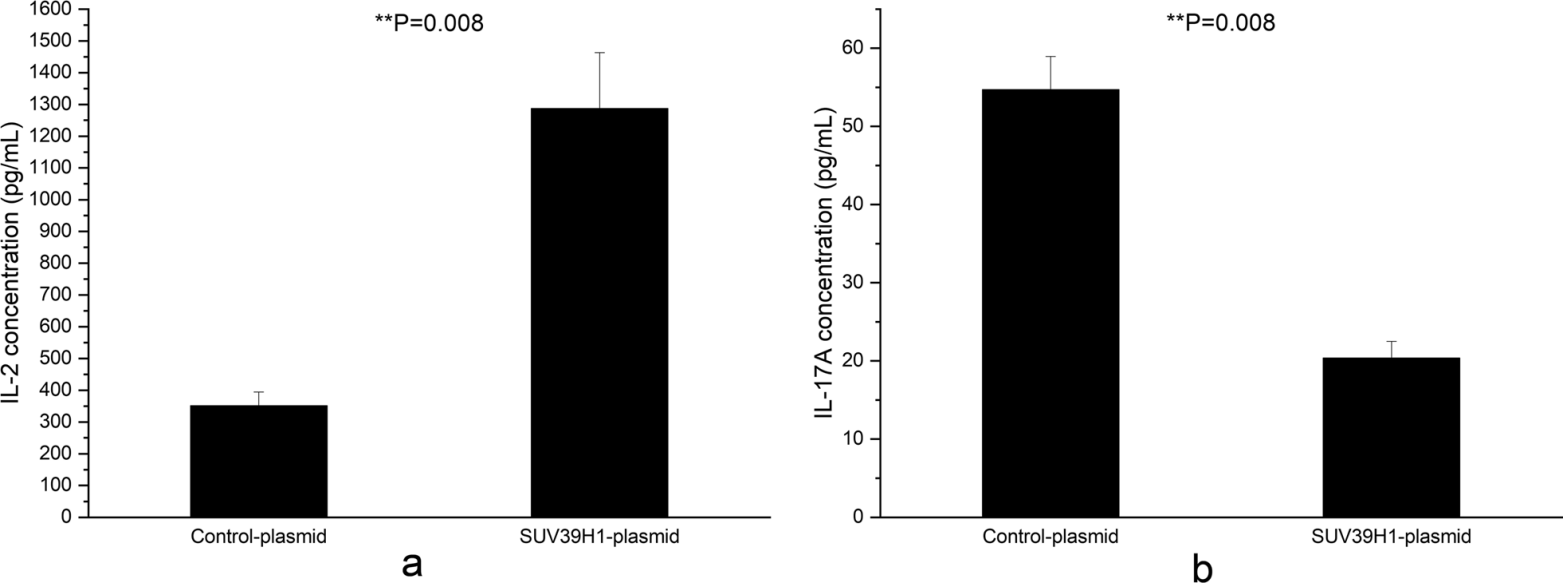
Effects of SUV39H1 up-regulation on IL-2 and IL-17A productions in CD4^+^ T cells from 3 SLE patients. **a**, **b** Relative IL-2 (**a**) and IL-17A (**b**) productions in the supernatants of SLE CD4^+^ T cells transfected with SUV39H1-plasmid or blank plasmid were detected by ELISA 72 h after transfection. All experiments were repeated three times

Next, the SUV39H1 overexpression plasmid (pcDEF3-SUV39H1) or blank plasmid (pcDEF3) was transfected into CD4^+^ T cells of 3 patients with SLE, and the cells and supernatant were harvested 72 h later. As expected, compared with CD4^+^ T cells transfected with the blank plasmid, the SUV39H1 protein expression (Fig. 5a, b), SUV39H1 (Fig. 5c) and H3K9me3 (Fig. 5d) enrichments in the CREMα promoter region of SLE CD4^+^ T cells transfected with the SUV39H1-overexpressing plasmid all strikingly upgraded, and the CREMα protein concentrations greatly alleviated (Fig. 5a, b). The abundances of DNA methylation (Fig. 5e) and DNMT3a (Fig. 5f) in the CREMα promoter region elevated intensely, while the H3K4me3 enrichment in the region decreased strikingly (Fig. 5g). No statistically significant difference was found in the change of Set1 binding (Fig. 5h). In the meantime, IL-2 over-secreted in the supernatant (Fig. 6a), while IL-17A was inhibited sharply (Fig. 6b).

### DNA methylation and DNMT3a levels were reduced, and H3K4me3 enrichment was elevated within CREMα promoter of SLE CD4^+^ T cells, and SUV39H1 binding positively correlated with the levels of DNA methylation and DNMT3a and negatively correlated with the H3K4me3 enrichment at this region

**Fig. 7:**
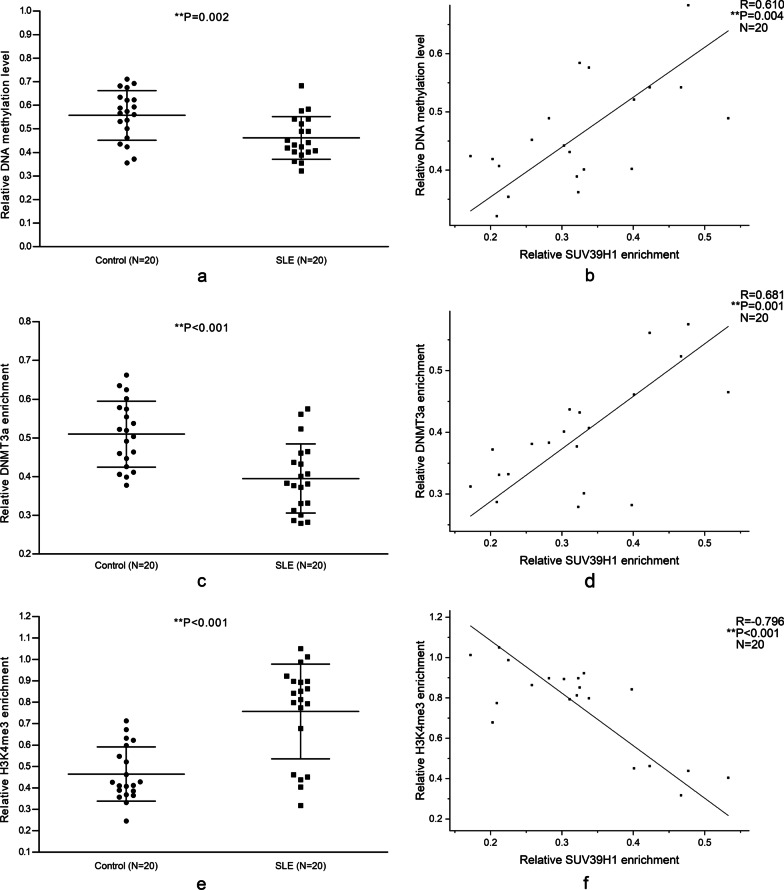
Relationships between SUV39H1 and DNA methylation, DNMT3a, and H3K4me3 at the CREMα promoter. **a** Relative DNA methylation level at the CREMα promoter in normal and SLE CD4^+^ T cells were examined by MeDIP combined with qPCR. **b** The correlation between SUV39H1 enrichment and DNA methylation level at the CREMα promoter in SLE CD4^+^ T cells. **c** Relative DNMT3a enrichment at the CREMα promoter in normal and SLE CD4^+^ T cells were examined by ChIP combined with qPCR. **d** The correlation between SUV39H1 enrichment and DNMT3a enrichment at the CREMα promoter in SLE CD4^+^ T cells. **e** Relative H3K4me3 enrichment at the CREMα promoter in normal and SLE CD4^+^ T cells were examined by ChIP combined with qPCR. **f** The correlation between SUV39H1 enrichment and H3K4me3 enrichment at the CREMα promoter in SLE CD4^+^ T cells. All reactions were performed in triplicate

Following the hint from the results of transfection, the level of DNA methylation at the CREMα promoter in the CD4^+^ T cells from the aforementioned 20 normal controls and 20 SLE patients was detected by MeDIP combined with qPCR. It is observed that the DNA methylation level in SLE patients was obviously lowered (Fig. 7a), and the SUV39H1 binding positively correlated with DNA methylation (Fig. 7b).

Zhang Q et al. [37] have proved that the DNMT3a enrichment is attenuated, and the H3K4me3 binding is elevated at the CREMα promoter of SLE CD4^+^ T cell. To further study the association between DNMT3a, H3K4me3, and SUV39H1 in the CREMα promoter region of SLE CD4^+^ T cells, the quantities of DNMT3a and H3K4me3 in the region of CD4^+^ T cells from the aforementioned cohort were measured by ChIP combined with qPCR. The data confirmed again that DNMT3a enrichment at the CREMα promoter was remarkably reduced in CD4^+^ T cells from SLE patients (Fig. 7c), and it was positively correlated with the SUV39H1 binding (Fig. 7d). Consistent with the finding of Zhang Q et al., we also determined the H3K4me3 level at the CREMα promoter of SLE CD4^+^ T cells was higher than normal controls (Fig. 7e), and it was negatively correlated with the SUV39H1 enrichment (Fig. 7f).

### Overexpressed Set1 binding at the CREMα promoter in SLE CD4^+^ T cells

**Fig. 8:**
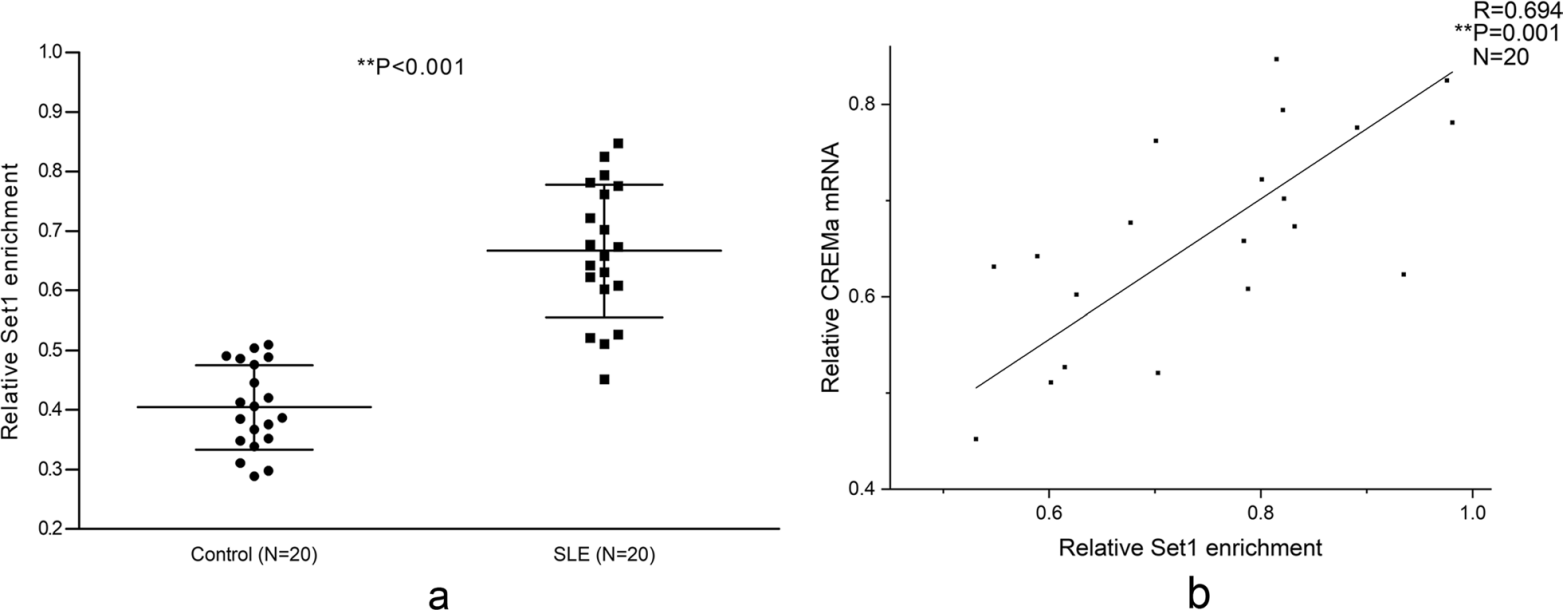
Set1 binding at the CREMα promoter in CD4^+^ T cells. **a** Relative Set1 binding at the CREMα promoter in normal and SLE CD4^+^ T cells was assessed by ChIP combined with qPCR. Input DNA was used as endogenous control, and IgG was used as negative control. **b** The correlation between Set1 binding at the CREMα promoter and CREMα mRNA expression in SLE CD4^+^ T cells. All experiments were repeated three times

Not only H3K9me3 can inhibit H3K4 methylation [31–33], but also H3K4me3 may down-modulate H3K9me3 level [41–43]. In order to get a more comprehensive view of how Set1-H3K4me3 involve in CREMα gene regulation and the relationship between Set1-H3K4me3 and SUV39H1-H3K9me3 axis, the Set1 binding at the CREMα promoter from the above-mentioned cohort was first examined. In line with the previous report [37], the Set1 enrichment at the CREMα promoter was remarkably higher in SLE CD4^+^ T cells than normal controls (Fig. 8a), and the Set1 enrichment was significantly positively correlated with the CREMα mRNA level in CD4^+^ T cells from SLE patients (Fig. 8b).

### Knocking down Set1 in SLE CD4^+^ T cells alleviated the CREMα promoter H3K4me3 enrichment, suppressed the expressions of CREMα and IL-17A, and elevated the levels of IL-2, CREMα promoter DNA methylation, and DNMT3a

**Fig. 9:**
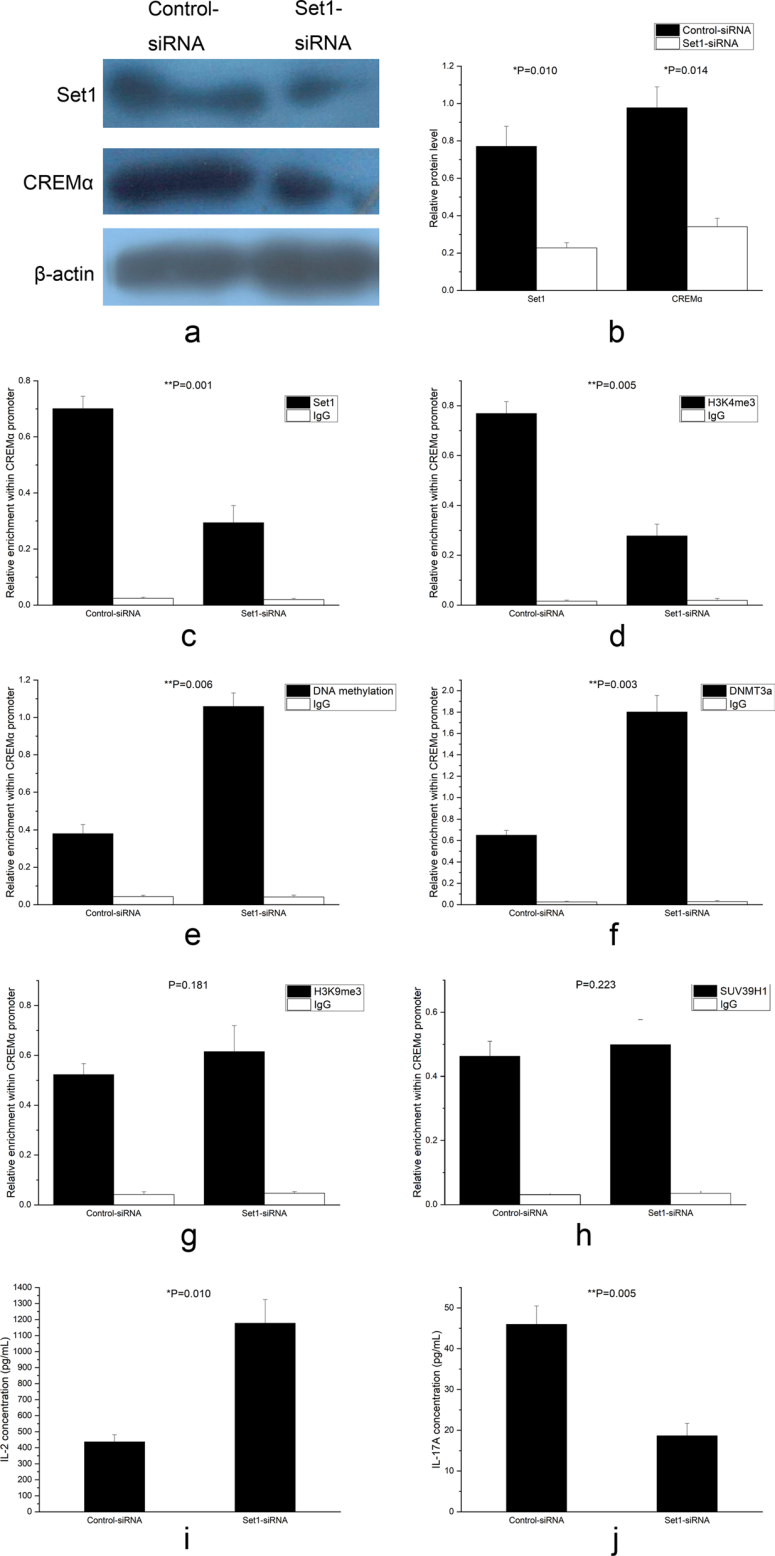
Effects of Set1 down-regulation on CD4^+^ T cells from 3 SLE patients. **a**, **b** Relative Set1 and CREMα protein expressions in SLE CD4^+^ T cells transfected with Set1-siRNA or control-siRNA were quantified by western blot analysis 72 h after transfection. β-actin was used as endogenous control. **c**, **d** Relative Set1 (**c**) and H3K4me3 (**d**) enrichments at the CREMα promoter in SLE CD4^+^ T cells transfected with Set1-siRNA or control-siRNA were analyzed by ChIP combined with qPCR 72 h after transfection. Input DNA was used as endogenous control, and IgG was used as negative control. **e**, **f**, **g**, **h** Relative levels of DNA methylation (**e**), DNMT3a (**f**), H3K9me3 (**g**), and SUV39H1 (**h**) at the CREMα promoter in SLE CD4^+^ T cells transfected with Set1-siRNA or control-siRNA were evaluated by ChIP or MeDIP combined with qPCR 72 h after transfection. Input DNA was used as endogenous control, and IgG was used as negative control. **i**, **j** Relative IL-2 (**i**) and IL-17A (**j**) productions in the supernatants of SLE CD4^+^ T cells transfected with Set1-siRNA or control-siRNA were detected by ELISA 72 h after transfection. All experiments were repeated three times

Set1 is the central element and catalytic subunit of complex of proteins associated with Set1 (COMPASS) [44–46]. Besides Set1, COMPASS contains seven other proteins [46]. Set1 alone has no activity to catalyze the methylation of H3K4, and it needs to function within COMPASS [37, 46]. Therefore, we transfected CD4^+^ T cells from 3 SLE patients with SUV39H1-siRNA or control-siRNA. After 72 h of transfection, the cells and supernatant were collected. The Set1 and CREMα proteins expressions were both markedly reduced in the Set1-siRNA group (Fig. 9a, b), In the meantime, the Set1 binding (Fig. 9c) and H3K4me3 enrichment (Fig. 9d) at the CREMα promoter also decreased greatly.

It has been reported that H3K4me3 is able to diminish DNA methylation by excluding DNMT3a [37, 47, 48]. Hence, we measured the levels of DNA methylation, DNMT3a, H3K9me3, and SUV39H1 at the CREMα promoter in the transfected CD4^+^ T cells. Compared with control-siRNA group, DNA methylation (Fig. 9e) and DNMT3a binding (Fig. 9f) at the CREMα promoter region in the Set1-siRNA group elevated obviously. However, there were no statistical differences in H3K9me3 (Fig. 9g) and SUV39H1 levels (Fig. 9h) within the region.

Then, the secretions of IL-2 and IL-17A were analyzed. The IL-2 concentration was higher strikingly in the supernatant of Set1-siRNA group (Fig. 9i), and the IL-17A quantity was attenuated dramatically (Fig. 9j).

## Discussion

As an immune-related factor, CREMα plays an important role in the onset and progression of SLE, especially in regulating the levels of IL-2 and IL-17A of T cells. Increased CREMα causes inhibition of IL-2 [3, 6, 10]. IL-2 is beneficial to improve the condition of SLE patients. The IL-2 concentration of SLE patients has a negative correlation with SLEDAI [2, 49]. The down-regulated IL-2 causes a deficiency in the body’s response to cytotoxins, and the weakened response to cytotoxins makes patients with SLE more vulnerable to infection. In addition, diminished IL-2 may suppress the number and function of Treg cells. Treg cells play a vital role in preventing autoimmunity, whose defect upgrades the autoimmune response. Moreover, reduced IL-2 also blocks activation-induced cell death (AICD). The decreased AICD prolongs survival period of autoreactive T cells, which leads to the continuous activation of B cells and excessive production of autoantibodies. All these ultimately promote the development of SLE [4, 50, 51].

Compared with IL-2, elevated CREMα induces IL-17A overproduction [2, 7, 8]. IL-17A has a positive correlation with SLE disease activity and anti-dsDNA titer in SLE patients. Reducing IL-17A alleviates the condition of patients with lupus [52–55]. As a pro-inflammatory factor, IL-17A leads to a wide range of inflammatory reactions, for instance, inducing various inflammatory mediators; recruiting monocytes, neutrophils, and T cells to invade target organs (such as kidney, blood vessels, skin, etc.); promoting the proliferation of B cells and production of antibodies (including total IgG, anti-DNA antibody, and anti-histone antibody), at last leading to tissue damage and disease development in SLE patients [52, 56, 57].

It has been confirmed that total T cells from SLE patients exhibit higher abundance of CREMα [2, 3]. However, there is no investigation about CREMα expression at the level of CD4^+^ T cells. By qPCR, we confirmed CREMα increased greatly in SLE CD4^+^ T cells for the first time ever. This result provided a foundation for our further research.

At present, the mechanism of CREMα over expression in SLE T cells has not been fully elucidated. Recently, more and more evidences show that epigenetics plays an important role in the pathogenesis of SLE. A series of epigenetic changes occur in the promoter region of immune-related genes in SLE patients [58–61]. Thus, the causes of CREMα change in terms of epigenetics were examined. H3K9me3 promotes DNA methylation [19, 20, 28–30] and inhibits H3K4 methylation [31–33]. Studies have confirmed that the DNA methylation level of the CREMα promoter region in total T cells from SLE patients is lower [2], while the H3K4me3 enrichment at the promoter in SLE CD4^+^ T cells is higher than that in normal controls [37]. Based on this, the H3K9me3 number in the CREMα promoter region of SLE CD4^+^ T cells was further examined by ChIP combined with qPCR, and it was first confirmed the H3K9me3 amount at the CREMα promoter was significantly lower in SLE CD4^+^ T cells compared to normal controls, and the H3K9me3 quantity negatively correlated with the CREMα expression and SLEDAI of SLE patients. These results strongly suggested that the reduction of H3K9me3 in the promoter region was one of the reasons for the overproduction of CREMα in SLE CD4^+^ T cells, and the H3K9me3 enrichment at the CREMα promoter could reflect the disease activity of SLE. Then, the cause of the down-modulated H3K9me3 enrichment was explored, revealing that H3K9 methyltransferase SUV39H1 in the CREMα promoter region of SLE CD4^+^ T cells alleviated greatly. The SUV39H1 binding in this region positively correlated with the H3K9me3 enrichment and negatively correlated with the CREMα level and SLEDAI score, but the SUV39H2 number had no significant difference between normal controls and patients with SLE. The results indicated that it was not SUV39H2, but SUV39H1 down-regulation in the CREMα promoter region of SLE CD4^+^ T cells that led to a reduction of H3K9me3 eventually promoted CREMα over expression and SLE progression.

SiRNA was used to inhibit the expression of SUV39H1 in normal control CD4^+^ T cells to verify the regulatory effect of SUV39H1 on CREMα. The result showed that SUV39H1 and H3K9me3 in the CREMα promoter region decreased, while the CREMα level upgraded. However, the plasmid-mediated overexpression of SUV39H1 in SLE CD4^+^ T cells had the opposite effect. These results demonstrated that SUV39H1 regulated CREMα quantity, and this regulation was at least partly achieved by changing the H3K9me3 enrichment in the CREMα promoter region.

The next question was whether the change in the SUV39H1 binding affected the numbers of DNA methylation and H3K4me3 at the CREMα promoter. The levels of DNA methylation, DNMT3a, H3K4me3, and Set1 in the CREMα promoter region of CD4^+^ T cells were further detected after transfection. The results showed that the abundances of DNA methylation and DNMT3a reduced, the H3K4me3 enrichment elevated, but the change in Set1 had no significant difference after down-regulating the SUV39H1 binding within the CREMα promoter in normal CD4^+^ T cells. At last, IL-2 production diminished, and IL-17A over-secreted in the supernatant of SUV39H1-siRNA group. Increasing the SUV39H1 number at the CREMα promoter in SLE CD4^+^ T cells had the opposite effects. These results suggest that decreased SUV39H1 and H3K9me3 in the CREMα promoter region of SLE CD4^+^ T cells might suppress the recruitment of DNMT3a, leading to reduction of DNA methylation. Interestingly, consistent with the results of this study, it has been confirmed that modulating the H3K9me3 enrichment at the promoter of some genes by regulating SUV39H1 leads to corresponding changes in the DNMT3a amount in this region [19, 21]. SUV39H1 also directly connects with DNMT3a and activates it through a conserved PHD-like motif [20, 21]. Moreover, HP-1 interacting with SUV39H1 directly connects and activates DNMT3a [20]. H3K9me3 also represses H3K4me3 by rejecting H3K4 methyltransferases or recruiting H3K4 demethylases [31–33]. The experimental results also confirmed that changing the H3K9me3 enrichment through SUV39H1 affected the H3K4me3 number, but this mechanism might not be achieved by altering the Set1 enrichment. Inhibition of DNA methylation and augment of H3K4me3 at the promoter further up-regulated CREMα, which affected the secretions of IL-2 and IL-17A, eventually leading to SLE. The transfection changed not only the SUV39H1 binding in the CREMα promoter region but also the overall SUV39H1 level; hence, the possibility that SUV39H1 regulated CREMα, IL-2, and IL-17A via other pathways could not be excluded.

Based on the results of transfection, the levels of DNA methylation, DNMT3a, and H3K4me3 in the CREMα promoter region of CD4^+^ T cells from the aforementioned subjects were detected. The data were consistent with the results of transfection: In SLE CD4^+^ T cells, the DNA methylation and DNMT3a binding were down-modulated, while H3K4me3 enrichment was increased greatly at the CREMα promoter. The SUV39H1 binding in the region positively correlated with the quantities of DNA methylation and DNMT3a and meanwhile negatively correlated with the H3K4me3 enrichment. These confirmed further that SUV39H1 might regulate the levels of DNMT3a, DNA methylation, and H3K4me3, affecting the CREMα expression.

It has been reported that the H3K4me3 and Set1 enrichments at the CREMα promoter were greatly elevated [37], and H3K9me3 and H3K4me3 are mutually exclusive at gene promoter regions [31–33, 41–43]. In our experiment, changing the SUV39H1 level did not affect the Set1 promoter binding; therefore, we studied if altering the Set1 amount could influence the H3K9me3 and SUV39H1 enrichments. We first confirmed that the Set1 number at the CREMα promoter of SLE CD4^+^ T cells was promoted, and it was positively correlated with the CREMα expression. These data proved Set1 played an important role in regulation of CREMα. Then by Set1-siRNA, we found reduced Set1 abundance alleviated H3K4me3 and augment the DNA methylation and DNMT3a enrichments within the CREMα promoter region of SLE CD4^+^ T cells, thus down-modulating the CREMα level, and augmenting IL-2 production and blocking IL-17A secretion. But there were no significant alters in H3K9me3 and SUV39H1 bindings at the CREMα promoter after transfection. These results suggested that in SLE CD4^+^ T cells, upgraded Set1 binding was able to elevate H3K4me3 level at the CREMα promoter. Increased H3K4me3 may antagonize DNMT3a, subsequently interfered with DNA methylation within the same region. At last, these factors facilitated CREMα overexpression, IL-2 deficiency, and IL-17A over-secretion, while this axis did not involve the SUV39H1-H3K9me3 axis.

Unlike classical genetics, epigenetic modifications are reversible. It has been proved that environment [62], food [63], and some medicines [64–67] can alter the epigenetic condition. According to our results, SUV39H1 could be a potential therapeutic target for SLE; therefore, we may find some kind of physical factors or medicines to promote it in SLE patients. Because SUV39H1 is an important histone methyltransferase that regulates a wide range of genes, we next step shall up-regulate it in SLE mouse model to verify its efficiency and side effect.

## Conclusions

**Fig. 10:**
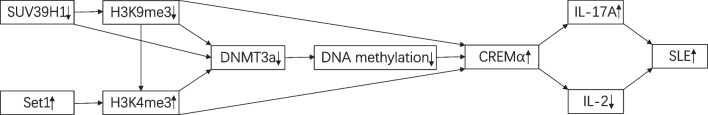
The regulation network and roles of SUV39H1-H3K9me3, Set1-H3K4me3, and DNMT3a-DNA methylation axes at the CREMα promoter of SLE CD4^+^ T cells

Our results show that the SUV39H1 binding in the CREMα promoter region of SLE CD4^+^ T cells alleviates, resulting in the diminished H3K9me3 enrichment. In the meantime, the Set1 amount at the CREMα promoter of SLE CD4^+^ T cells is up-regulated. Decreased H3K9me3 and increased Set1 elevate the H3K4me3 number and repress the DNMT3a production; moreover, deficient SUV39H1 may attenuate the DNMT3a recruitment directly, whereafter inhibit DNA methylation. All of these promote CREMα transcription and production of IL-17A and interfere with secretion of IL-2 in CD4^+^ T cells, ultimately promoting the onset and development of SLE (Fig. 10). Our research detects the CREMα quantity at the level of CD4^+^ T cells, reveals the role that SUV39H1 plays in CREMα regulation, and elucidates the correlations between SUV39H1-H3K9me3, Set1-H3K4me3, and DNMT3a-DNA methylation at the CREMα promoter of SLE CD4^+^ T cells for the first time ever. These will provide a new idea for treating SLE.

## Data Availability

The datasets used and/or analyzed during the current study are available from the corresponding author on reasonable request.

## Abbreviations

SLE: Systemic lupus erythematosus
CREMα: cAMP-responsive element modulator α
SLEDAI: Systemic lupus erythematosus disease activity index
H3K9me3: Histone H3 lysine 9 trimethylation
H3K4me3: Histone H3 lysine 4 trimethylation
HP-1: Heterochromatin protein-1
SUV39H1: Suppressor of variation 3–9 homolog 1
SUV39H2: Suppressor of variation 3–9 homolog 2
HMTs: Histone methyltransferases
qPCR: Quantitative PCR
ChIP: Chromatin immunoprecipitation
DNMT3a: DNA methyltransferase 3a
Set1: SET domain containing 1
ACR: American College of Rheumatology
cDNA: Complementary DNA
ELISA: Enzyme-linked immunosorbent assays
MeDIP: Methylated DNA immunoprecipitation
COMPASS: Complex of proteins associated with Set1
AICD: Activation-induced cell death
